# Green Tea Polyphenol EGCG Attenuates MDSCs-mediated Immunosuppression through Canonical and Non-Canonical Pathways in a 4T1 Murine Breast Cancer Model

**DOI:** 10.3390/nu12041042

**Published:** 2020-04-10

**Authors:** Ping Xu, Feng Yan, Yueling Zhao, Xiangbo Chen, Shili Sun, Yuefei Wang, Le Ying

**Affiliations:** 1 Department of Tea Science, Zhejiang University, Hangzhou 310058, China; zdxp@zju.edu.cn (P.X.); 11616050@zju.edu.cn (Y.Z.); zdcy@zju.edu.cn (Y.W.); 2 Australian Centre for Blood Diseases, Central Clinical School, Monash University, Melbourne 3004, Australia; feng.yan@monash.edu; 3 Key Laboratory of Molecular Epigenetics of the Ministry of Education, Northeast Normal University, Changchun 130024, China; chensunbor@126.com; 4 Tea Research Institute, Guangdong Academy of Agricultural Sciences, Guangzhou 510640, China; sunshili@zju.edu.cn; 5 Centre for Innate Immunity and Infectious Diseases, Hudson Institute of Medical Research, Clayton 3168, Australia

**Keywords:** EGCG, anti-tumor mechanism, MDSCs, immunosuppression, non-canonical pathways

## Abstract

Several studies in the past decades have reported anti-tumor activity of the bioactive compounds extracted from tea leaves, with a focus on the compound epigallocatechin-3-gallate (EGCG). However, further investigations are required to unravel the underlying mechanisms behind the anti-tumor activity of EGCG. In this study, we demonstrate that EGCG significantly inhibits the growth of 4T1 breast cancer cells *in vitro* and *in vivo*. EGCG ameliorated immunosuppression by significantly decreasing the accumulation of myeloid-derived suppressor cells (MDSCs) and increasing the proportions of CD4^+^ and CD8^+^ T cells in spleen and tumor sites in 4T1 breast tumor-bearing mice. Surprisingly, a low dose of EGCG (0.5–5 μg/mL) effectively reduced the cell viability and increased the apoptosis rate of MDSCs *in vitro*. EGCG down-regulated the canonical pathways in MDSCs, mainly through the Arg-1/iNOS/Nox2/NF-κB/STAT3 signaling pathway. Moreover, transcriptomic analysis suggested that EGCG also affected the non-canonical pathways in MDSCs, such as ECM–receptor interaction and focal adhesion. qRT-PCR further validated that EGCG restored nine key genes in MDSCs, including *Cxcl3*, *Vcan*, *Col4a1*, *Col8a1*, *Oasl2*, *Mmp12*, *Met*, *Itsnl* and *Acot1*. Our results provide new insight into the mechanism of EGCG-associated key pathways/genes in MDSCs in the murine breast tumor model.

## 1. Introduction

Tea (*Camellia sinensis*) has become one of the most consumed beverages in the world, not only because of its special flavor, but also for its potential benefits to human health, such as preventing cancers [1,2]. Epigallocatechin-3-gallate (EGCG) is a fundamental polyphenol compound present in green tea, which was first reported in 1987 to exhibit anti-tumor effects. Since this report, EGCG has gained a lot of attention in the research community [3]. In recent decades, numerous studies have investigated the role of EGCG’s anti-tumor function which have suggested that ECGC plays a role in effectively retarding the growth and progression of tumors *in vitro* and *in vivo*, even in some human clinical trials [2,3]. Proposed possible mechanisms behind this anti-tumor function include cellular proliferation and apoptosis, modulation of cellular signaling pathways, inhibition of the growth of blood vessels and attenuation of oxidative DNA damage [4]. However, the mechanisms behind the anti-tumor function of ECGC remain elusive because of the poor bioavailability of ECGC *in vivo*, and the effective concentration of ECGC *in vitro* (> 20 μM) could not be reached in animals or humans [5,6,7].

Tumor initiation, growth and metastasis are promoted by immunosuppression mediated by immunosuppressive cells. Among them, myeloid-derived suppressor cells (MDSCs) are found to be abnormally expanded in various types of tumors in mice and humans, and mainly contribute to the negative regulation of anti-tumor immune responses [8,9]. MDSCs mediate immunosuppression primarily through the canonical mechanisms, by increasing the expression of Arginase 1 (Arg-1) and the production of reactive oxygen species (ROS) and nitric oxide (NO), as well as some cytokines (such as IL-6, IL-10 and TGF-β), resulting in impaired T-cell immune response [10,11].

Abundant evidence shows that EGCG possesses potent antioxidant and remarkable anti-inflammation activities. Interestingly, the effect of Polyphenon E (a commercial product that is mainly comprised of EGCG) on anti-tumor immune response was investigated by Santilli et al. [12]. Polyphenon E inhibited neuroblastoma growth *in vivo* by promoting differentiation of MDSCs to more neutrophilic form through 67 kDa laminin receptor and the induction of colony-stimulating factor (G-CSF) [12]. This study provided new insight to understand the anti-tumor mechanism of EGCG. However, whether EGCG can inhibit MDSC-mediated immunosuppression in other cancer models, and what sorts of key genes and pathways are involved in MDSCs, are yet to be further illustrated.

Metastatic breast cancer is a prominent cause of death among women worldwide [13,14]. Eliminating MDSCs enhances anti-tumor immune response and improves the efficacy of breast cancer therapies [15,16]. The murine 4T1 breast cancer model was employed as a classic model to study MDSCs [17]. In the present study, *in vitro* and *in vivo* models were used to evaluate the potential inhibitory effects of EGCG on 4T1 breast cancer cells. We further assessed the changes of several key immune cells in the tumor microenvironment, including MDSCs and T cells (CD4^+^ T and CD8^+^ T cells) in blood, spleen and tumor in the 4T1 tumor-bearing mice. Moreover, the regulatory impact of EGCG on MDSCs were investigated *in vitro*, and the key genes and pathways that EGCG could restore in MDSCs were further explored in this study.

## 2. Materials and Methods 

### 2.1. Materials and Reagents

EGCG (purity > 98%) was purchased from Huzhou Rongkai Foliage Extract Company (Huzhou, China). Antibodies used in this study were: anti-β actin (Proteintech Group, Chicago, IL, USA); anti-NF-κB, anti-phospho-NF-κB, anti-STAT3, anti-phospho-STAT3 (Cell Signaling Technology, Danvers, MA, USA). All other chemicals were analytical grade and purchased from Shanghai Boer Chemical Reagent Company (Shanghai, China).

### 2.2. 4T1 Cell Culture and In Vitro Assays

The 4T1 breast cancer cell line was purchased from American Type Culture Collection (ATCC). The 4T1 cells were maintained in RPMI-1640 medium (Gibco, Carlsbad, CA, USA) supplemented with 10% fetal bovine serum (FBS) (Gibco, Carlsbad, CA, USA), 100 U/mL of penicillin and 100 U/ml of streptomycin (Gibco, Carlsbad, CA, USA) at 37 °C in an incubator containing 5% CO_2_.

CCK8 assay (Biosharp, Hefei, China) was used to study the cell viability. A total of 1 × 10^4^ cells were seeded into a 96-well plate in 100 μL RPMI-1640 medium. For detection, 10 μL CCK8 reagent was added into each well and absorbance at 450 nm was recorded after 1-h incubation in dark.

Wound healing assay and transwell assay were applied according to the protocols used in our previous studies [18,19]. Wound healing assay was used to assess the migration ability of 4T1 cells. Briefly, 5 × 10^5^ cells were seeded into a 6-well plate and treated with different concentrations of EGCG. 4T1 cells were scratched with a sterile 200 μL tip and monitored for the following 48 h. Representative images were taken at 24 h and 48 h after scratching. Tranwell assay was applied to detect cell invasion ability. Transwell chambers (Corning, Rochester, NY, USA) were put into a 24-well transwell plate. Twenty percent FBS in cell medium was added into the lower chamber, and 5 × 10^5^ 4T1 cells were seeded in the upper well. After treatment with different EGCG for 24 h, the images on the lower side of the insert filter were taken under microscope. The invasion ability (relative invasion percentage) was calculated by counting three different fields of cells adhering to the lower surface of the transwell system.

### 2.3. Animal Study

Male, 6-week-old BALB/c mice were purchased from Shanghai SLAC Laboratory Animal Company (Shanghai, China). All mice were housed under specific pathogen-free (SPF) conditions. All the experiments were performed according to protocols approved by the Zhejiang University Institutional Animal Care and Use Committee (ZJU20190004). Mice were randomly divided into six groups (*n* = 5): two groups given sterile water and four groups administered water supplemented with different doses of EGCG (250 μg/mL, 500 μg/mL, 1000 μg/mL and 2000 μg/mL, respectively) throughout the experiment. After one-month pretreatment, except for one group as a healthy/negative control group, the remaining five groups were injected with 1 × 10^6^ 4T1 cells subcutaneously. The group without treatment of EGCG was considered as a positive control. All mice were scarified on day 21 after inoculation of 4T1 cells, and the blood, spleens and tumors of mice were collected for further experiments.

### 2.4. Flow Cytometry Analysis

Flow cytometry was conducted using an ACEA NovoCyteTM (ACEA Biosciences, San Diego, CA, USA) device. For detecting the apoptosis rate of cells, the annexin V-FITC/PI kit was purchased from Dojindo Company (Shanghai, China). 1 × 10^6^ MDSCs were seeded into a 6-well plate. After EGCG treatments for the indicated time, cells were stained with annexin V and PI, and then analyzed by flow cytometry.

For cell cycle detection, 1 × 10^6^ MDSCs were treated with different concentrations of EGCG. Pre-cold 70% ethanol was used to fix the cells at 4 °C overnight. Before staining, cells were washed with PBS buffer and adjusted to 200 μL. Then, 100 μL of RNase A was added to the cell suspension and incubated at 37 °C for 30 min. 400 μL PI dye was mixed into the cell suspension, and the mixture was incubated at at 4 °C for 30 min before flow cytometry analysis.

For assessing the proportion of immune cells, single cell suspensions were washed with PBS buffer. 1 × 10^6^ cells from blood, spleen and tumor were prepared to obtain single-cell suspension. After centrifugation at 1500 rpm, 4^o^C for 5 min, 3 mL red blood cell lysing buffer (Sigma-Aldrich, St. Louis, MO, USA) were added to the cells from blood and spleen. Cells were vortexed for 30 s and left on ice for 5 min. After that, 15 mL cold PBS was added to neutralize the solution. Anti-CD45-APC, anti-CD11b-PE, anti-Gr-1-FITC, anti-CD4-FITC, anti-CD8-PE (eBioscience, San Diego, CA, USA) were used for staining procedures.

### 2.5. Cell Sorting and MDSCs In Vitro Experiments

Male, 6-week-old BALB/c were injected with 1 × 10^6^ 4T1 cells subcutaneously and were sacrificed for cell sorting after two weeks of injection. MDSCs were isolated from mice spleen by magnetic activated cell sorting using MACS MicroBeads, MACS separation columns and SuperMACS II Separator (Miltenyi Biotec, Bergisch Gladbach, Germany). Briefly, 1 × 10^8^ spleen cells were labeled by Biotin anti-mouse Ly-6G/Ly-6C (Gr-1) antibody (Biolegend, San Diego, CA, USA) and MACS Microbeads (Miltenyi Biotec, Bergisch Gladbach, Germany). After rinsing the column with degassed buffer (provided with columns from Miltenyi Biotec, Bergisch Gladbach, Germany), the cell suspension was applied onto the column. The magnetically labeled cells were collected for the downstream *in vitro* experiments. 1 × 10^6^ of MDSCs were seeded into 12-well plates and treated with either control (medium) or different concentrations of EGCG for 6 h. After that, supernatants were collected for cytokine detection and cells were harvested for either RNA or protein extraction.

### 2.6. RNA Isolation and Quantitative Reverse Transcription PCR (qRT-PCR)

Total RNA was extracted using Trizol Reagent (Takara, Shiga, Japan). qRT-PCR was conducted on a CFX-Touch real-time PCR machine (Bio-Rad, Hercules, CA, USA) using SYBR Green reagent (Roche, Basel, Switzerland). All primer sequences are listed in Supplementary Table S1.

### 2.7. Western Blot

Cells were lysed in cold RIPA buffer (Solarbio Science & Technology, Beijing, China) supplemented with 1 mM PMSF (Solarbio Science & Technology, Beijing, China) and 10 mM phosphatase inhibitor (Solarbio Science & Technology, China). The cell lysates were centrifuged at 12,000× *g* for 10 min at 4 °C to remove the precipitate. Protein concentrations of samples were examined using a BCA protein assay kit (Beyotime Biotechnology, Nantong, China). Equal amounts of protein were separated by SDS-PAGE and transferred to PVDF membranes (Millipore, Billerica, MA, USA). Membranes were blocked with 5% skim milk in TBST for 1 h at room temperature and incubated with primary antibodies overnight at 4 °C. After primary antibody incubation, the membranes were rinsed three times in TBST followed by incubation with HRP-labeled goat anti-rabbit IgG antibody (Servicebio, Wuhan, China) for 1 h at room temperature. β-Actin was used as a loading control.

### 2.8. Detection of Arginase Activity, NO and ROS Production

Cell lysates were collected for measuring arginase activity using QuantiChrom arginase assay kit (BioAssay Systems, Hayward, CA, USA). Nitrites were measured in culture supernatants using Greiss reaction kit (Invitrogen, Shanghai, China). For determination of ROS production, we used Reactive Oxygen Species Assay Kit (Beyotime Biotechnology, Nantong, China), and fluorescence intensity of DCFDA was evaluated by flow cytometry.

### 2.9. RNA Sequencing and Microarray Data Analysis

MDSCs were treated with either control medium or 5 μg/mL EGCG *in vitro* for 6 h. After that, RNA was extracted and sent for RNA sequencing (RNA-seq). Raw sequencing data in FASTQ format was aligned to GRCm38 (release 91) using STAR (2.6.0c) with default setting and was deposited on GEO under accession number: GSE135685. Gene count matrix was generated using featureCounts (1.6.2) with Ensembl annotation (release 91) [20]. Differential expression (DE) analysis was performed using edgeR (3.24.3) with quasi-likelihood test, and DE genes were filtered with false discovery rate (FDR) < 0.05 [21]. Public microarray data was obtained from GSE39807 using GEOquery (2.50.5) [22], with RMA normalization and log2 transformation. DE analysis was performed using limma (3.38.3) linear model and empirical Bayes-moderated t-statistics [23]. DE genes were filtered with |log Fold Change| > 1 and FDR < 0.05. Kyoto Encyclopaedia of Genes and Genomes (KEGG) pathway analysis was performed using clusterProfiler (3.10.1) [24]. Heatmap was generated using log count per million for RNA-seq and log-normalized intensity for microarray with the pheatmap package (1.0.12). All these analyses were done in R 3.5.0. Gene set enrichment analysis (GSEA) was performed using GSEA (4.0) software [25]. The input gene expression matrix was log2-transformed counts per million from the raw RNA-seq count matrix. The input phenotype information was 2 EGCG-treated and 2 control samples. The input gene sets including MDSC survival [26,27,28] and expansion [29] gene sets were derived from previous publications. Default settings were used except for permuting gene sets instead of samples due to the limited sample size. 

### 2.10. Statistical Analysis

Statistical analysis was done using GraphPad Prism 7 software; all data are shown as mean ± SEM and were analyzed using the one-way analysis of variance (one-way ANOVA) test by GraphPad Prism 7. Differences were considered significant when *p* < 0.05.

## 3. Results

### 3.1. EGCG Inhibits 4T1 Tumor Growth Both In Vitro and In Vivo

**Figure 1:**
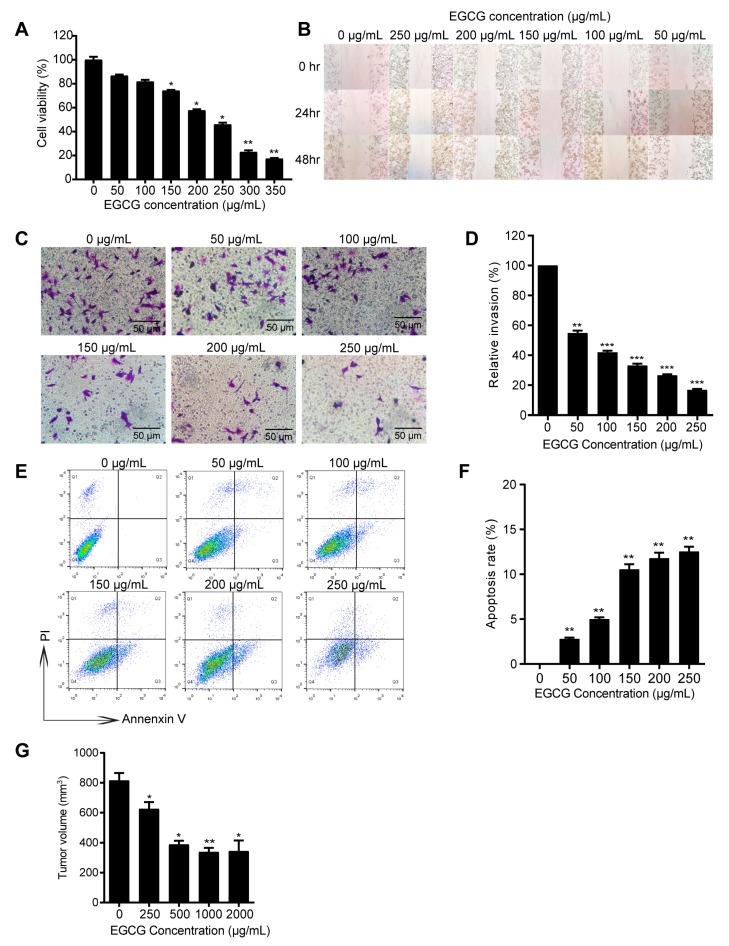
Inhibitory effect of epigallocatechin-3-gallate (EGCG) on 4T1 tumor cells *in vitro* and *in vivo* (**A**). The cell viability of 4T1 cells significantly decreased after 24-h treatments of EGCG (150–350 μg/mL) compared to the control group. (**B**). For wound healing assay, 4T1 cells were wounded by scratching and monitored over 24 and 48 h. (**C**). The invasion abilities of 4T1 cells were compared by different doses of EGCG treatments after 24 h. (**D**). The relative invasion ability of 4T1 cells was significantly decreased in EGCG treatment groups (50–250 μg/mL) compared to the control group. (**E**). Representative plots of apoptosis analysis by flow cytometry after 24-h treatment of EGCG. (**F**). Significantly higher apoptosis rate was observed in EGCG treatment groups (50–250 μg/mL) compared to the untreated group. (**G**). Tumor volumes in EGCG (250–2000 μg/mL) treatment groups were significantly reduced compared to the control group. In each group, the number of mice was 5. Data are presented as mean ± SEM. *, *p* < 0.05; **, *p* < 0.01; ***, *p* < 0.001.

Firstly, 4T1 cells were treated with different concentrations of EGCG for 24 h *in vitro*, then the inhibitory effect of EGCG on 4T1 cells was evaluated by CCK8 assay. As shown in Figure 1A–F, EGCG not only remarkably suppressed the cell viability (effective EGCG concentration from 150–350 μg/mL), migration and invasion of 4T1 cells (effective EGCG concentration from 50–250 μg/mL), but also dose-dependently induced the apoptosis of 4T1 cells (effective EGCG concentration from 50–250 μg/mL). Furthermore, to assess the anti-tumor activity of EGCG *in vivo*, mice were treated with different concentrations of EGCG in drinking water one month before injection of 4T1 cells. Mice were sacrificed 21 days after the 4T1 cell injection. The tumor growth in EGCG treated mice was significantly inhibited compared to the control group (without EGCG treatment) (Figure 1G). The effective concentrations of EGCG *in vitro* were above 50 μg/mL, whereas the *in vivo* concentration needed to suppress 4T1 cells is very low (250 μg/mL drinking water, equivalent to around 0.5 μg/mL in mice plasma due to low bioavailability) [30]. This suggests that other factors could be involved in EGCG-anti-tumor effect *in vivo*.

### 3.2. EGCG Ameliorates MDSCs-Mediated Immunosuppression in 4T1 Tumor-Bearing Mice

**Figure 2:**
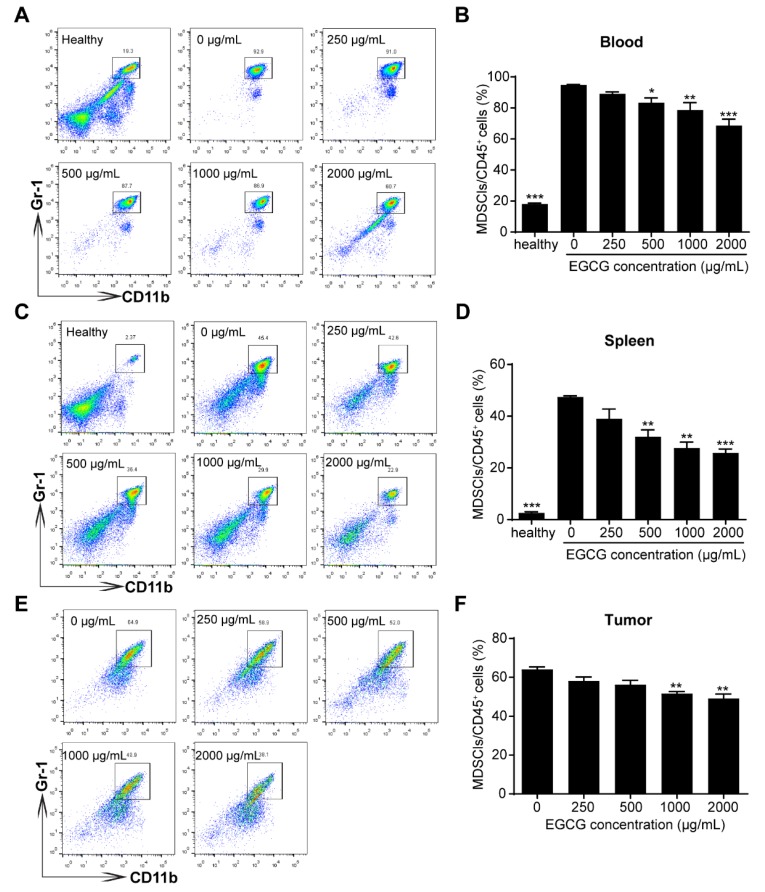
EGCG suppresses the accumulation of myeloid-derived suppressor cells (MDSCs) in the blood, spleen and tumor in 4T1 breast tumor-bearing mice. Decreased MDSC percentages in blood (**A**–**B**), spleens (**C**–**D**) and tumors (**E**–**F**) in mice (*n* = 5) were observed after treatments with different EGCG concentrations, which were analyzed by flow cytometry. The representative plots (left) and bar plots (right) are shown. Data are presented as mean ± SEM for 5 mice per group. *, *p* < 0.05; **, *p* < 0.01; ***, *p* < 0.001.

As there were possibly other factors in the tumor microenvironment involved in EGCG-mediated anti-tumor effects, we further investigated the proportions of MDSCs and T cells (both CD4^+^ and CD8^+^ T cells) in the peripheral blood, spleen and tumor tissues, to see whether these cells were influenced by the EGCG treatment. MDSCs (CD45.2^+^CD11b^+^Gr-1^+^) strongly accumulated in peripheral bloods and spleens in 4T1 tumor-bearing mice compared to that of healthy mice (Figure 2A–D). Interestingly, EGCG treatments significantly reduced the percentages of MDSCs in the peripheral bloods and spleens (≥ 500 μg/mL) (Figure 2A–D), and in tumor sites (≥1000 μg/mL) (Figure 2E,F).

**Figure 3:**
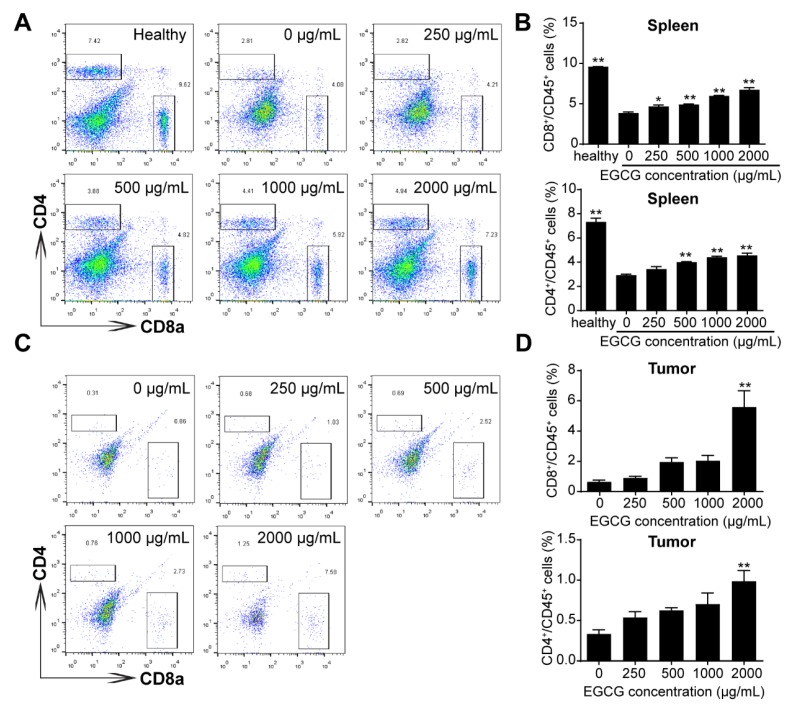
EGCG increases the proportion of CD4^+^ and CD8^+^ T cell in the spleen and tumor in 4T1 tumor-bearing mice. The percentages of CD4^+^ and CD8^+^ T cell of spleens (**A**–**B**) and tumors (**C**–**D)** from healthy and 4T1 tumor-bearing mice (*n* = 5) were analyzed by flow cytometry. The representative plots (left) and bar plots (right) are shown. Data are presented as mean ± SEM. *, *p* < 0.05; **, *p* < 0.01.

Furthermore, administration of EGCG was able to partly restore the percentages of CD4^+^ T helper cells and CD8^+^ cytotoxic T cells in the spleen and tumor tissues (Figure 3A–D), indicating the anti-tumor immune response in tumor-bearing mice is enhanced by EGCG treatments.

### 3.3. EGCG Suppresses Growth and Increases Apoptosis of MDSCs In Vitro

**Figure 4:**
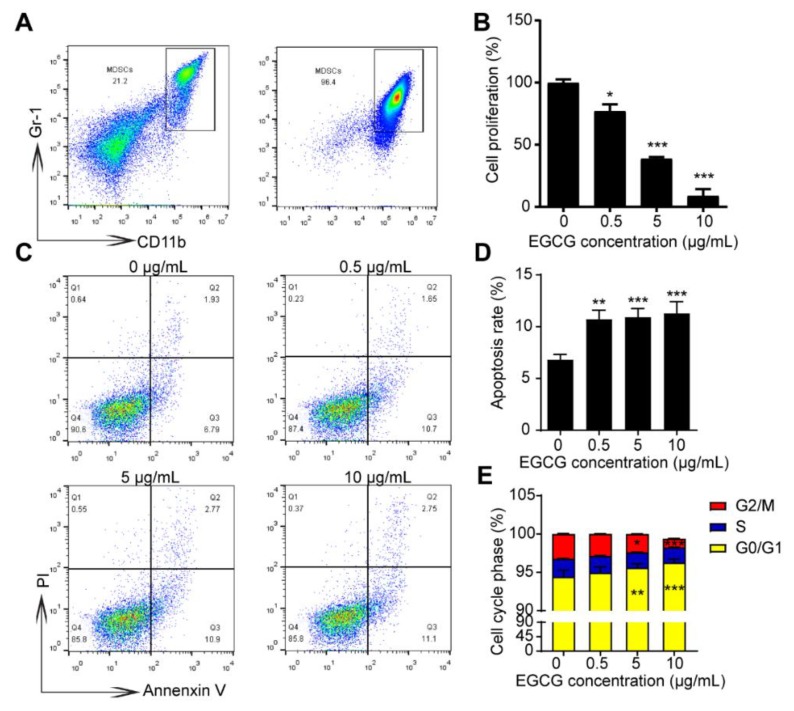
EGCG reduces cell viability and increases the apoptosis rate of MDSCs *in vitro*. (**A**) Representative plots showing the MDSCs from the spleen of 4T1 tumor-bearing mice before and after sorting. (**B**) The cell viability of MDSCs was significantly reduced by the indicated concentrations of EGCG for 6 h compared to control group. The effects of EGCG on the cell viability of MDSCs were assessed by CCK8 assay. (**C**) Apoptosis rate of MDSCs significantly increased after 6 h-EGCG treatment. Representative plots (**C**) and statistical charts (**D**) are shown. (**E**) Cell-cycle analysis of MDSCs after 6 h-EGCG treatment is shown. Data are presented as mean ± SEM. *, *p* < 0.05; **, *p* < 0.01; ***, *p* < 0.001.

To confirm whether MDSCs could be affected by EGCG directly, MDSCs were isolated and purified from spleens of 4T1 tumor-bearing mice using magnetic activated bead sorting. As indicated in Figure 4A, we could get around 96% of MDSCs in CD45^+^ cells compared to only 21% before sorting. Impressively, a strong suppression of the MDSCs’ cell viability was observed at the intermediate EGCG concentration (5 μg/mL); at a relatively higher concentration (10 μg/mL) of EGCG, the cell viability of MDSCs decreased to ~10% (Figure 4B), suggesting a strong inhibitory effect of EGCG on the survival of MDSCs. EGCG treatments (from 0.5 μg/mL to 10 μg/mL) significantly (*p* < 0.05) increased the apoptosis rate of MDSCs (Figure 4C-D). MDSCs were arrested at G0/G1 phases upon EGCG treatment at the concentration ≥ 5 μg/mL (Figure 4E). Notably, EGCG was able to inhibit the survival and induce the apoptosis of 4T1 cells at such low concentrations (0.5, 5 and 10 μg/mL) (Figure S1), implying a possibility that EGCG may target MDSCs to inhibit the growth of tumor cells.

**Figure 5:**
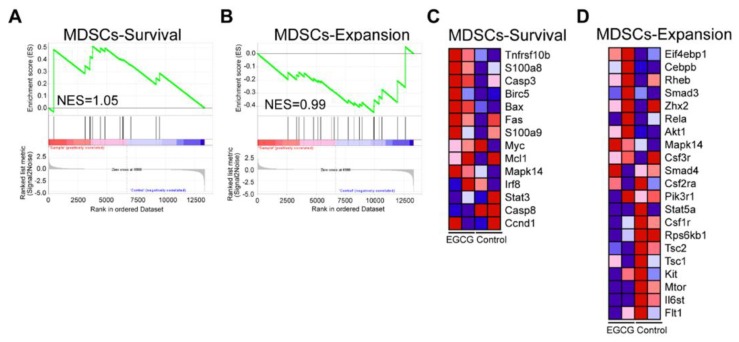
Gene set enrichment analysis (GSEA) results of MDSC survival and expansion gene sets after treatment of EGCG based on RNA-seq data. MDSC survival (**A**) and MDSC expansion gene sets (**B**) were enriched after 6 h-EGCG treatment. A heatmap of the key genes in MDSC survival (**C**) and MDSC expansion (**D**) is displayed. Red color indicates high expression and blue color indicates low expression.

We further sorted the MDSCs and treated them with either control or EGCG (5 μg/mL) for 6 h *in vitro*, and then the cells were harvested for RNA-seq. GSEA analysis showed enrichment of MDSC survival and expansion gene sets (normalized enrichment scores (NES) were 1.05 and 0.99 respectively), suggesting that EGCG treatments could alter the survival and expansion of MDSCs (Figure 5A,B). EGCG could inhibit the survival of MDSCs by increasing the expression of the key genes induced cell apoptosis/death in MDSCs (Figure 5C). The majority of genes involved in MDSCs expansion gene sets showed decreased expression upon EGCG treatment compared to the control group (Figure 5D), suggesting an inhibitory effect of EGCG on the expansion of MDSCs. Collectively, these results indicate that EGCG could efficiently suppress the survival and expansion of MDSCs according to the GESA analysis.

### 3.4. EGCG Targets MDSCs through the Canonical Pathways

**Figure 6:**
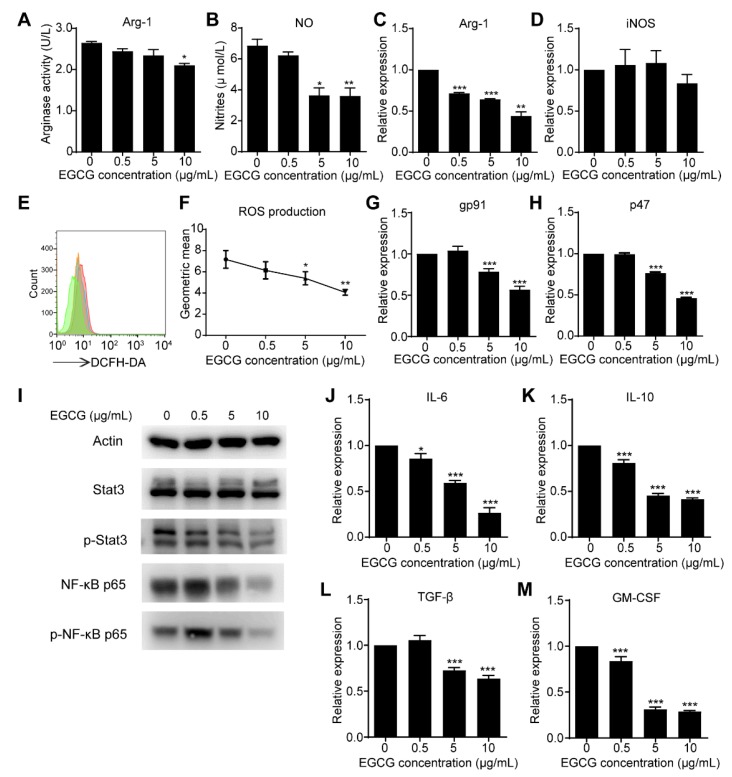
Inhibitory effects of EGCG on canonical pathways in MDSCs *in vitro*. Activity of Arg-1 (**A**) and concentration of NO (**B**) in supernatant significantly decreased in EGCG treatments compared to control group. (**C**) The mRNA levels of *Arg-1* were significantly reduced in MDSCs after EGCG treatment (**D**). No significant difference was found between EGCG and control groups on the mRNA levels of *iNOS.* (**E**,**F**) The production of ROS was reduced by EGCG treatments. (**G**,**H**) The mRNA levels of *p47-phox* and *gp91-phox* in MDSCs significantly decreased when treated with 5–10 μg/mL of EGCG. (**I**) The phosphorylated NF-κB and STAT3 were determined by Western blot. The mRNA levels of *IL-6* (**J**), *IL-10* (**K**), *TGF-β* **(**L**)**, *GM-CSF* (**M**) decreased in MDSCs after 6 h-EGCG treatment. Data are presented as mean ± SEM. *, *p* < 0.05; **, *p* < 0.01; ***, *p* < 0.001.

The activation of MDSCs leads to the up-regulation of immune suppressive factors such as Arg-1, iNOS, NO and ROS [10,11]. To further understand how EGCG regulates MDSCs, these factors were investigated. As exhibited, EGCG was able to dampen the activity of Arg-1 and the production of NO in the supernatant of MDSCs after 6 h treatment (Figure 6A,B). qRT-PCR results showed the RNA expression of *Arg-1* decreased accordingly, but no significant difference was observed in the expression of *iNOS* (Figure 6C,D) compared to the control group. Meanwhile, ROS production was also reduced by EGCG treatments (Figure 6E,F). STAT3 activation is directly responsible for upregulating the transcription of NADPH oxidase 2 (Nox2) subunits, neutrophil cytosol factor 1 (p47-phox) and NADPH oxidase subunit gp91-phox in MDSCs [11]. Further investigations showed the expression of *p47-phox* and *gp91-phox* was significantly reduced by EGCG treatments (Figure 6G,H), and the phosphorylation of STAT3, not STAT3, was remarkably downregulated accordingly based on the Western blot results (Figure 6I). NF-κB is a well-known transcription factor, and its phosphorylation directly regulates the expression of Arg-1, iNOS and other cytokines [31,32]. EGCG was shown to downregulate NF-κB p65 and phosphorylated NF-κB p65 (Figure 6I) in a dose-dependent manner in MDSCs. Since EGCG was able to inhibit the activation of NF-κB signal pathway (Figure 6I), its down-regulation on the expression of cytokines, especially for *IL-6*, *IL-10*, *TGF-β* and *GM-CSF* was further validated in MDSCs accordingly (Figure 6J–M).

In addition, *nuclear factor erythroid 2-related factor 2* (*Nrf2*) and *Nrf2*-related antioxidant protein OH-1 play a crucial role in defensing cellular stress [33], and endogenous antioxidant enzymes, such as catalase (CAT), superoxide dismutase (SOD) and glutathione-S- transferase (GST) [34]. However, no significant differences in these genes in EGCG treatment groups were observed when compared to the control group (Figure S2). Collectively, EGCG could target MDSCs via NF-κB/pSTAT3 canonical pathways.

### 3.5. EGCG Restores MDSCs through Non-Canonical Signaling Pathways

**Figure 7:**
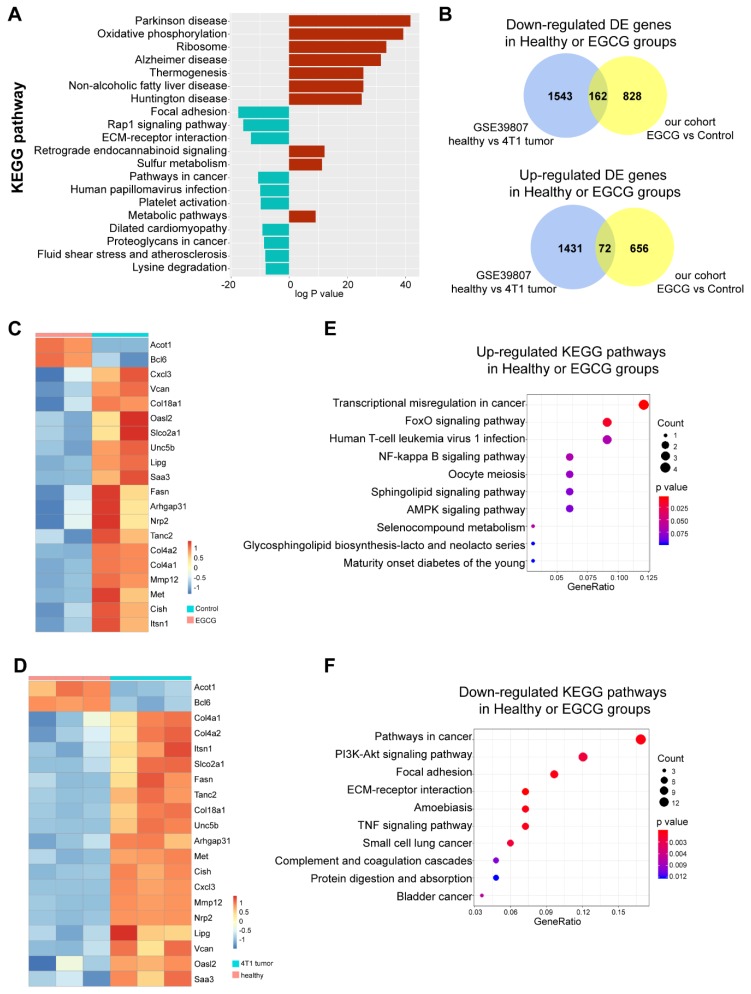
EGCG restores key genes and pathways in MDSCs. (**A**). Results from Kyoto Encyclopaedia of Genes and Genomes (KEGG) pathway analysis. The top 20 pathways up- (red color) and down-regulated (blue color) in EGCG treatment groups are shown. (**B**). Venn-diagrams of the up-regulated and down-regulated differential expressed (DE) genes in our cohort and GSE39807 are shown. The middle area showed the overlapping genes. (**C**,**D**). Expressions of 20 top overlapped DE genes are shown in the heatmap. Based on hierarchical clustering, a clear separation can be observed between Control and EGCG groups and between the 4T1 tumor-bearing mice and the healthy mice. (**E**,**F**). The top 10 up-/down-regulated KEGG pathways enriched in the shared DE genes are shown.

Having demonstrated that EGCG could efficiently suppress the survival of MDSCs, we further investigated the novel/non-canonical pathways that EGCG affected. Based on the RNA-seq data, we were able to characterize that Parkinson’s disease, oxidative phosphorylation, ribosomes, Alzheimer’s disease, and thermogenesis were the top 5 up-regulated pathways in the EGCG treatment group, while focal adhesion, the Rap1 signaling pathway, extra cellular matrix (ECM)–receptor interaction, pathways in cancer and human papillomavirus infection were the top five down-regulated pathways in EGCG treatment group (Figure 7A).

**Figure 8:**
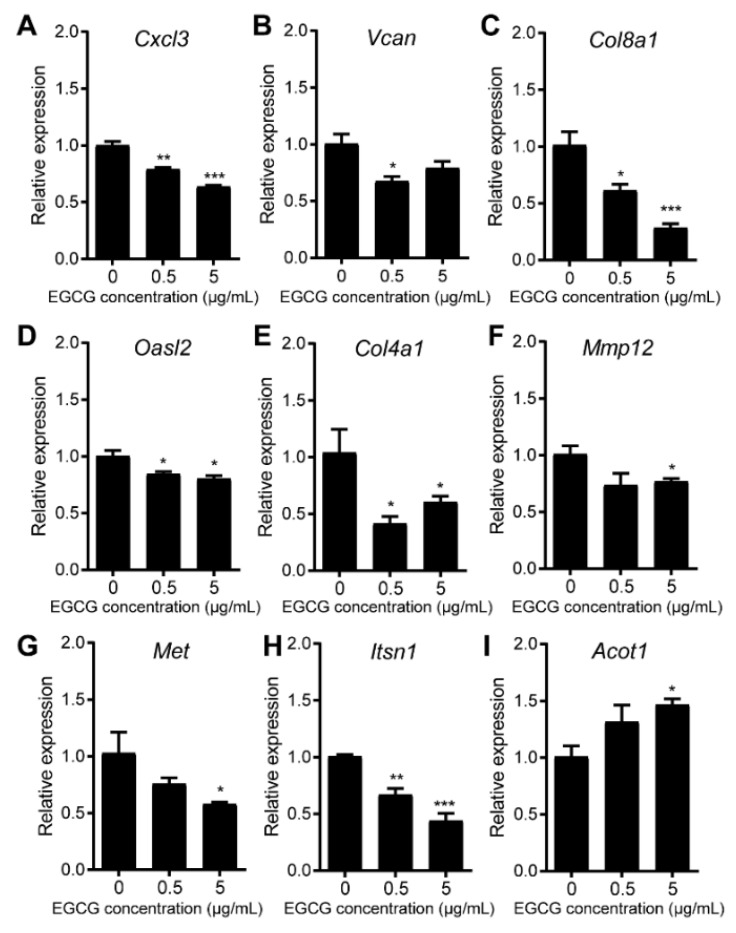
The mRNA expression changes in eight genes in MDSCs are validated by qRT-PCR. The relative mRNA expression of *Cxcl3* (**A**), *Vcan* (**B**), *Col8a1* (**C**), *Oasl2* (**D**), *Col4a1* (**E**), *Mmp12* (**F**), *Met* (**G**), *Itsn1* (**H**), and *Acot1* (**I**). MDSCs were treated with 0, 0.5 and 5 μg/mL of EGCG for 6 h. Data are presented as mean ± SEM. *, *p* < 0.05; **, *p* < 0.01; ***, *p* < 0.001.

To further identify the key genes that EGCG restored in MDSCs, we integrated our sequencing data with the dataset GSE39807 [22], which was the microarray data of CD11b^+^ MDSCs sorted from healthy and 4T1 tumor-bearing BALB/c mice. There were 162 shared down-regulated genes and 72 shared up-regulated genes in healthy or EGCG treatment groups compared to untreated tumor groups (Figure 7B). We further filtered the top 20 differentially expressed (DE) genes shared by EGCG treatments and healthy group. The heatmap results clearly show that in healthy and EGCG treated groups *B-cell lymphoma 6 (Bcl6)* and *Acyl-CoA thioesterase 1 (Acot1)* were significantly up-regulated, while *Chemokine (C-X-C motif) ligand 3 (Cxcl3), Versican (Vcan), Collagen type IV alpha 1 chain (Col4a1), Collagen type IV alpha 2 chain (Col4a2), Collagen Type XVIII Alpha 1 chain (Col18a1), 2’-5’ oligoadenylate synthetase-like 2 (Oasl2), solute carrier organic anion transporter family member 2A1 (Slco2a1), unc-5 netrin receptor B (Unc5b), lipase G, endothelial type (Lipg), serum amyloid A 3 (Saa3), fatty acid synthase (Fasn), Rho GTPase activating protein 31 (Arhgap31), neuropilin 2 (Nrp2), tetratricopeptide repeat, ankyrin repeat and coiled-coil containing 2 (Tanc2), matrix metallopeptidase 12 (Mmp12), MET proto-oncogene, receptor tyrosine kinase (Met), cytokine inducible SH2 containing protein (Cish)* and *intersectin 1* (*Itsn1)* were down-regulated (Figure 7C,D). We further validated the expression levels in MDSCs by qRT-PCR. The changes of these nine candidate genes, namely, *Cxcl3, Vcan, Col8a1, Oasl2, Col4a1, Mmp12, Met, Itsnl* and *Acot1*, were consistent with the RNA-seq results (Figure 8). For the rest of the 12 genes, the RNA expression of eight genes did not change after treatment with EGCG (Figure S3), while four of them showed the opposite changes after EGCG treatments (Figure S4), when compared to the RNA-seq results.

Among the nine genes that showed consistent changes in both RNA-seq results and qRT-PCR results, *Cxcl3* and *Mmp12* have been reported to relate to the cell migration ability [35], survival/suppressive activity [36], and the accumulation of MDSCs [37] in different cancer models, while the functions of the other genes in MDSCs have not been reported yet. 

Next, to check the key pathways that EGCG restored in MDSCs, KEGG pathway analysis was further utilized to unveil the top down- or up-regulated pathways in MDSCs in the EGCG treatment group and healthy BALB/c mice, compared to the untreated tumor group. As shown in Figure 7E, transcriptional misregulation in cancer, FoxO signaling pathway, human T-cell leukemia virus 1 infection, NF-κB signaling pathways and oocyte meiosis were the top five up-regulated KEGG pathways in the EGCG treatment and healthy BALB/c groups. Moreover, the top five down-regulated pathways in the EGCG treatment and healthy BALB/c groups were pathways in cancer, PI3K-Akt signaling pathway, focal adhesion, ECM–receptor interaction and amoebiasis (Figure 7F).

## 4. Discussion

In the present study, we found that EGCG could effectively inhibit the growth of 4T1 breast cancer cells both *in vitro* and *in vivo.* However, the effective EGCG concentration *in vitro* is above 50 μg/mL, which cannot be reached in the plasma of mice/humans. This phenomenon has facilitated further studies of the impact of EGCG on MDSCs cells. It has been reported that EGCG inactivated the MDSCs in a previous neuroblastoma model by Santili et al. [12]. We further validated that EGCG could also significantly reduce the percentage of MDSCs in blood, spleen and tumor sites in 4T1 tumor-bearing mice model, suggesting that EGCG may play a similar impact on MDSCs across different types of tumors. Moreover, *in vitro* results indicated that EGCG significantly suppressed the survival of MDSCs and induced the apoptosis rate of MDSCs. We confirmed that EGCG could target canonical pathways in MDSCs, by decreasing the expression of phosphorylated STAT3 (pSTAT3) and NF-κB p65. Additionally, we further investigated the other non-canonical pathways and novel genes that EGCG regulated in MDSCs by RNA sequencing. The RNA-seq data showed that EGCG could target several novel pathways including ECM–receptor interaction, focal adhesion and the PI3K-Akt signaling pathway in MDSCs. Importantly, EGCG could restore the expression of nine key genes in MDSCs, which was validated by qRT-PCR. For seven out of nine genes, it was shown for the first time that EGCG could target these genes in MDSCs.

An array of evidence demonstrates that EGCG has an inhibitory ability on various types of tumors [1,3,4]. Our results further confirm the effective anti-tumor effect of EGCG in 4T1 breast tumor model both *in vitro* and *in vivo*. However, the molecular characteristics of EGCG (molecular weight 458 g/mol and eight phenolic groups in its chemical structure) limit its bioavailability according to Lipinski’s rule of five [38]. According to the previous studies, the maximum plasma concentration of EGCG in humans reached 7.5 μM (3.4 μg/mL) when treated by oral dose (equivalent 16.9 mg/kg) of EGCG [39]. When mice were treated with high oral doses (2000 mg/kg) of EGCG, peak plasma concentrations of ~9 μM (4.1 μg/mL) were observed [30]. Usually, the effective concentrations of EGCG acting against tumor *in vitro* were higher than 20 μM (9.2 μg/mL), as is also supported in our study (≥50 ug/mL) [40,41]. 

Regarding the 4T1 breast tumor model in the present study, our results suggested that the effective dose of EGCG was above 50 μg/mL *in vitro* (Figure 1), which is much higher than the effective concentration in plasma of mice or humans. Therefore, additional factors in the tumor microenvironment should be considered. MDSC-mediated immunosuppression is involved in initiation, growth and metastasis of various types of tumors including breast tumor, and MDSC is considered a promising target for tumor immunotherapy [42,43]. It was found that EGCG could attenuate MDSC-mediated immunosuppression in tumor-bearing mice by decreasing the accumulation of MDSCs in peripheral blood, spleen, and tumor tissues (Figure 2). Proportion of T cells (both CD4^+^ and CD8^+^ T cells), as the main targets of MDSCs, were increased accordingly with decreased MDSCs proportion (Figure 3). These results indicate that the anti-tumor immune response of tumor-bearing mice had been enhanced by EGCG, resulting in the inhibition of tumor growth. Similar effects of EGCG on MDSCs have also been reported in a neuroblastoma model [12].

Santili and colleagues used a commercial product called ‘polyphenon E’ to study the role of EGCG on MDSCs in this neuroblastoma model [12]. The equivalent dose of EGCG in their study was ~5–10 μg/mL *in vitro* and ~3000 mg/kg *in vivo*. Meanwhile, in this study, we used 0.5–5 μg/mL for the *in vitro* experiment and 250–2000 mg/kg for the *in vivo* experiment. Interestingly, we observed that effective EGCG concentration was around 0.5–5 μg/mL *in vitro* and around 500–1000 mg/kg *in vivo*. MDSCs were found to be very sensitive to EGCG treatment *in vitro*. EGCG significantly inhibited the growth of MDSCs at an extremely low concentration (0.5 μg/mL, approximately equivalent to 1 μM) (Figure 4); this concentration could be easily reached in the plasma of mice based on the previous literature [6,7,30]. These results suggest EGCG can efficiently suppress the growth and expansion of MDSCs (Figure 4 and Figure 5).

The EGCG dose we used in this study was slightly lower compared to the equivalent dose of EGCG used by Santili et al [12]. This may have several explanations. Firstly, the effective dose of EGCG in 4T1 tumor models may be lower than that of the neuroblastoma models. Secondly, as a commercial product, polyphenon E consists 53% epigallocatechin 3-gallate (EGCG), 9% epicatechin, 11% (-)-epigallocatechin, 5% epicatechin-3-gallate, and 5% (-)-gallocatechin gallate, which is a mixture of different catechins [12]. It was not clear whether the mixture of these compounds exhibited better bioactivities compared to a single EGCG treatment. Thirdly, Santili and colleagues did not mention whether they had tried lower concentrations of polyphenon E. Lower doses of EGCG might also work in the neuroblastoma model.

The canonical suppressive features of MDSCs are characterized by elevated levels of Arg-1 activity and iNOS, associated with increased production of NO and ROS in the supernatants, which contribute to the suppression of T-cell function [9,10]. In the present study, EGCG was demonstrated to target MDSCs by the canonical pathways, mainly through Arg-1/iNOS/Nox2 (*gp91* and *p47*)/ NF-κB /STAT3 signaling (Figure 6). In addition, MDSCs are one of the main host cells contributing to the immune suppressive microenvironment, where immunosuppression is mainly enhanced by cellular crosstalk mediated by cytokines [44]. EGCGs significantly suppress such crosstalk via inhibiting the expression of mediators in MDSCs (Figure 6). These results explain the observation that anti-tumor immunity was enhanced further by EGCG treatment in 4T1 tumor-bearing mice.

In addition to the canonical pathways, we further investigated the novel key genes and pathways that EGCG could potentially impact in MDSCs by RNA sequencing. We filtered the top 20 genes that EGCG were able to restore in MDSCs, and nine genes were further validated by qRT-PCR analysis. Among these, *Cxcl3* and *Mmp12* have been closely linked to the survival and function of MDSCs [35,36,37]. Versican, encoded by the *Vcan* gene, was reported to impact the accumulation of MDSCs, and silencing Versican promoted the anti-tumor efficacy of endostatin in a B16F1 melanoma tumor model [45]. However, the remaining six genes have not been linked to roles in MDSCs, which could suggest that they are potential novel targets in MDSCs by EGCG treatments.

*Col4a1* and *Col8a1* are collagen genes, which are highly related to collagen chain trimerization [46]. Intriguingly, our previous studies showed that EGCG treatment shared four common collagen-related genes *Col1a1*, *Col1a2*, *Col3a1* and *Col6a3* with atorvastatin treatment in non-alcoholic fatty liver diseases [47]. This may suggest a potential role of EGCG on the collagen–ECM–receptor interaction pathways across different diseases.

*Oasl2* is a gene involved in Interferon gamma (IFN-γ) signaling, cytokine signaling and PI3K-Akt signaling pathways [48]. *Oasl2* exhibits a key role in mediating interferon production and antiviral infection [49]; however, there is currently no evidence for its role in cancer. *Met* [50], *Itsn1* [51], and *Acot1* [52] all show a pivotal role in tumor development; however, the role of these genes in MDSCs is currently unknown. Further investigations of the key genes within these pathways are crucial to unveiling potential key targets for ECGC against MDSCs. 

Meanwhile, EGCG could down-regulate focal adhesion and ECM–receptor interaction in MDSCs (Figure 7). Focal adhesions act as the mechanical linkages to the ECM; therefore, EGCG may play a role in the interaction and structure between cells. 

Recently, immunotherapy has become a powerful clinical strategy for cancer therapy [53,54,55]. Levels of MDSCs, NO and ROS in the peripheral blood are critical biomarkers for monitoring the immune system response and the overall success of immunotherapy [56]. Pretreatment through the suppression of the immune system by neutralizing chronic inflammation and/or directly inhibiting MDSCs has been shown to increase the efficacy of immunotherapy [57]. Future studies may reveal if EGCG has a potential application as a MDSCs inhibitor for cancer adjuvant immunotherapy.

## 5. Conclusions

In conclusion, our results demonstrate that EGCG could impact the survival and apoptosis rate of MDSCs in a 4T1 breast cancer model. EGCG could not only target the canonical signaling pathways and reduce the production of related cytokines in MDSCs, but also affect some non-canonical signaling pathways such as ECM–receptor interaction and focal adhesion. Nine key genes that are restored by EGCG in MDSCs may provide new insight into the inhibitory effects of EGCG on MDSCs and the potential mechanism of action on MDSCs in the 4T1 breast cancer model.

## Supplementary Materials

The following are available online at https://www.mdpi.com/2072-6643/12/4/1042/s1, Figure S1: The effects of EGCG on cell proliferation and apoptosis of 4T1 at low concentrations. Figure S2: The mRNA levels of *Nrf2*, *OH-1* and endogenous antioxidant enzymes in MDSCs. Figure S3: The mRNA expression of eight genes in MDSCs treated with EGCG (0.5 or 5 μg/mL) did not change when compared to control (0 μg/mL). Figure S4: The mRNA expression of *Saa3*, *Fasn* and *Cish* in MDSCs treated with EGCG (5 μg/mL) significantly increased when compared to control (0 μg/mL). Table S1: qRT-PCR primer sequences.
